# Bruton's tyrosine kinase: oncotarget in myeloma

**DOI:** 10.18632/oncotarget.655

**Published:** 2012-09-12

**Authors:** Yu-Tzu Tai, Kenneth C Anderson

**Affiliations:** LeBow Institute for Myeloma Therapeutics and Jerome Lipper Multiple Myeloma Center, Dana-Farber Cancer Institute, Harvard Medical School, Boston, MA, USA; LeBow Institute for Myeloma Therapeutics and Jerome Lipper Multiple Myeloma Center, Dana-Farber Cancer Institute, Harvard Medical School, Boston, MA, USA

Targeting Bruton's tyrosine kinase (Btk), an essential element of B cell receptor (BCR) signaling pathway, has achieved remarkable efficacy with an acceptable safety profile in B cell malignancies. Ibrutinib (formally PCI-32765) is an orally available inhibitor with excellent pharmacodyamics, which irreversibly and selectively binds to Btk, since the cysteine residue (Cys481) to which it binds is present in only 10 of >500 kinases in the human genome. It has achieved high response rates in phase I/II clinical trials in relapsed non Hodgkin's lymphoma, and phase III clinial trials in mantle cell lymphoma and chronic lymphocytic leukaemia (CLL) are ongoing for FDA approval. Due to its efficacy and lack of toxicity, it has great promise, either as a single agent or in combination, in other B cell malignancies as well.[1]

As normal B cells mature to plasma cells, they lose surface immunoglobulin, as is true for multiple myeloma (MM) cells. Therefore, B cell receptor signaling and Btk expression on normal plasma cells and MM cells is unexpected.[2] However, our recent studies using gene expression profiling and immunoblotting demonstrated robust Btk expression in the majority (>85%) of patient MM cells and in all tumor cells from patients with Waldenstrom's macroglobulinemia (WM).[3] Importantly, Ibrutinib effectively blocked both baseline and induced activation of Btk and downstream NFκB, STAT3, ERK1/2, and Akt signaling pathways mediating tumor cell proliferation and survival. Specifically, Ibrutinib-induced inactivation of Btk and downstream NFκB and STAT3 is correlated with tumor cell cytotoxicity. Intriguingly, Btk is highly expressed and activated in dexamethasone (dex)-resistant MM1R MM cells, but not in parental MM1S cells that are dex sensitive, suggesting a role for Btk in drug resistance. Our studies further show that WM cells express CD19 and BCR, which mediate growth and survival, whereas MM cells lack both, accounting, at least in part, for the greater direct cytotoxicity of Ibrutinib against WM than MM cells. Nonetheless, shBtk knockdown in 3 Btk-expressing MM cell lines, including MM1R cells, confirmed that Btk also directly regulates MM cell viability.

**Figure 1:**
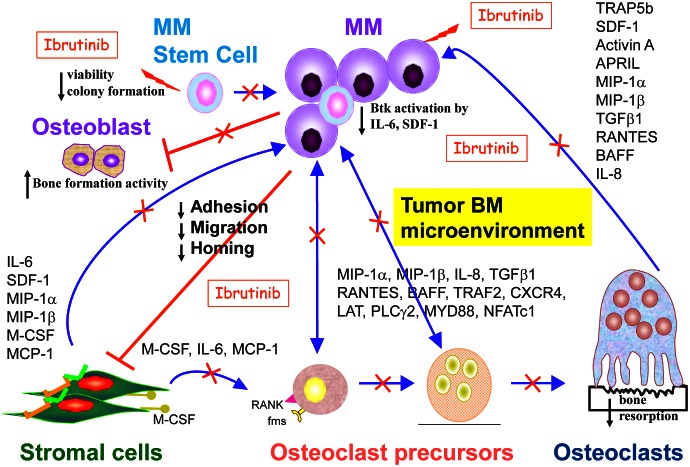
Btk inhibition by Ibrutinib/PCI-32765 in MM Functional sequelae following Btk inhibition indicates that Ibrutinib not only target tumor cells but also bone marrow microenvironment that support MM cell growth and survival, as well as MM-deteriorated bone lysis. These results demonstrate Btk inhibitors are extremely attractive approach for the novel treatment of myeloma and related bone disease. Data adapted.[3]

Importantly, we also found that Ibrutinib is most potent against IL-6- or stromal-dependent MM cells in coculture with patient-derived bone marrow stromal cells (BMSCs) or osteoclasts (OCs), suggesting that that Ibrutinib-mediated cytotoxicity against MM cells was indirect via targeting the MM BM microenvironment. Indeed, Ibrutinib strongly reduced secretion of multiple cytokines and chemokines in MM cocultures with BMSCs, including IL-6, SDF-1, activin A, MIP-1α, BAFF, IL-8, and M-CSF (Figure 1). In particular, Ibrutinib decreased MIP1 and MIP-1β excretion in MM and WM cells, as well as OCs. Of note, in our in vitro models, Ibrutinib specifically blocked OC formation from osteoclast precursor cells and bone resorption, without affecting bone formation. These results are consistent with previous reports of Btk expression in OC, but not in osteoblasts (OB), in genetically manipulated mice.[4] In addition, Ibrutinib blocked SDF-1 secretion from BM accesory cells, as well as SDF-1-induced Btk activation and migration in MM cells. Similar results were also recently reported in CLL, which might partially explain significant clinical responses.[5]

Most importantly, Ibrutinib suppressed tumor growth and improved MM-induced bone lysis in our SCID-hu mice model, in which MM cells implanted into human bone chips both grow and cause bone lesions. Not only was OC number and function reduced, but osteoblastogenesis was also increased, evidenced by greater alkaline phosphatase (ALP) activity (>1-log difference), in Ibrutinib versus control-treated animals. Thus, by inhibiting MM cell growth and survival in vivo, Ibrutinib relieves the inhibition of new bone formation by MM cells. Indeed our studies do confirm that slightly increased bone mineral density in Ibrutinib-treated versus control mice. These findings strongly support a novel therapeutic strategy to target Btk and thereby improve osteolytic bone disease in MM, analogous to therapies targeting Btk to maintain cartilage integrity in autoimmune arthritis.[6]

Our findings therefore provide a strong rationale for investigating Btk inhibitors in MM and WM to target both tumor cells and their supporting BM microenvironment and thereby both suppress tumor cell growth and abrogate MM-induced bone disease. Finally, the recently identified MyD88 L265P mutation in 90% WM cells promotes survival of WM cells by activation of Btk,[7, 8] along with Ibrutinib-inhibited IRF4 activity induced by MyD88 mutation in diffuse large B cell lymphoma[1] further suggest its clinical promise in a variety of B cell malignancies.
